# GABA Receptor mutant *gbb-1* accelerates morphological aging of GABA neurons in *Caenorhabditis elegans*

**DOI:** 10.17912/D7E5-WJ67

**Published:** 2018-09-06

**Authors:** I. Dhillon, A. Momin, I. Chin-Sang

**Affiliations:** 1 Queen’s University, Kingston, ON, Canada

**Figure 1.**
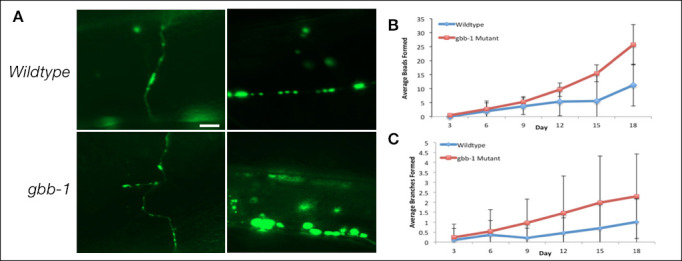


## Description

The aging of an organism is heterogeneous, that is to say, tissues and organs may age at unique rates compared to overall organism longevity. Inhibition of the insulin/insulin-like growth factor signaling (IIS), such as the *daf-2* IIS receptor increases *Caenorhabditis elegans* longevity*.* The *daf-2* pathway plays a key role in the regulation of metabolism and stress responses and as such, impacts the longevity of the organism (Uno and Nishida 2016). Evidence suggests that an increase in insulin signaling results in a reduction in overall lifespan and a decrease in neuronal longevity (Kenyon 2010). Recently, the *gbb-1* GABA signaling pathway has also been implicated in the regulation of adult longevity (Chun *et al.* 2015). The GABAergic motor neurons innervate the dorsal and ventral body muscles controlling locomotion, foraging and defecation of the animal. GABA, an inhibitory neurotransmitter, has been found to decrease the lifespan of the nematodes (Chun *et al.* 2015), however, its role in neuronal aging has yet to be determined. The formation of ectopic neurite branches and production of axonal beads are two known phenotypes of neuronal aging in *C. elegans.* In this study, we examined the aging of the GABAergic motor neuron nerve cords and dorsal commissures.

Fifty age-synchronized worms were imaged every three days (days 3, 6, 9, 12, 15, 18). Worms were plated on Fluorodeoxyuridine (FUdR) treated agar plates in order to maintain only adult worms throughout the imaging process (Mitchell *et al.* 1979). Neurons were visible using the GABAergic specific *unc-25::GFP* marker strain. To keep the data consistent, images were taken of the GABAergic axons and dorsal commissures between the VD5 and DD5 cell bodies (Panel A, scale bar 10 microns). Fluorescent images were taken using the Zeiss Axioplan fluorescent microscope under 40x objective. To prepare slides for fluorescence microscopy, an agarose immobilization protocol was followed (Fang-Yen *et al.* 2009). Aging of the neurons was quantified by counting the number of axonal beads and branches present on the neural processes. Branched processes emanating from the cell body, neural axon or dorsal commissures were scored as aging phenotypes. As predicted, GABAergic neurons displayed phenotypic evidence of aging.

The *C. elegans* genome encodes two metabotropic GABAB receptor genes, *gbb-1* and *gbb-2,* which are homologous to their mammalian counterparts (Chun *et al.* 2015)*.* GABA binds to the *gbb-1* receptor initiating a signaling cascade that ultimately inhibits the *daf-16* transcription factor. A reduction of function mutation in the *gbb-1* encoded GABA receptor subunit increases worm lifespan; however, it seems to be specific to the GBB-1 subunit as no effect was observed in a loss of function *gbb-2* mutation. When overexpressed, *gbb-1* mutants were short-lived which is consistent with GABA signaling regulating lifespan (Chun *et al.* 2015). To determine the effect of GABA signaling on neuronal aging, we observed the effect of a loss-of-function *gbb-1* mutant on the aging of the GABAergic motor neurons. Unexpectedly, the GABAergic dorsal commissures and axons in the *gbb-1* mutants displayed a significant increase in aging morphology compared to the wild type (Panel A). *gbb-1* mutants displayed a significant increase in beading (Panel B, Table 1) and branching (Panel C, Table 2) in the GABAergic motor neurons compared to the wild type from day 9 onwards (*p<*0.05, independent t-test, n=50). Thus *gbb-1(tm1406)* mutants although long lived, have neurons that appear to age faster. This work was done with a single *gbb-1* allele, in the future it will be important to verify our findings with additional *gbb-1* alleles or show that a transgene with the wildtype *gbb-1* can suppress the observed aging phenotypes.

**Table 1.** Average bead formation on GABAergic dorsal commissures and axons between DD5 and VD5 cell bodies in N2 wild type and *gbb-1* mutant worms (n=50).

**Table:** 

**Day**	**3**	**6**	**9**	**12**	**15**	**18**
***wild type***	0.06 ± 0.24	1.88 ± 2.87	3.70 ± 1.94	5.36 ± 2.42	5.56 ± 3.01	11.26 ± 7.23
***gbb-1* mutant**	0.50 ± 1.05	2.7 ± 3.01	5.24 ± 2.95	9.62 ± 5.06	15.46 ± 9.43	25.7 ± 7.49
**p-value**	0.005^*^	0.166	0.003^*^	5.27 x 10^-7*^	2.26 x 10^-10*^	3.12 x 10^-16*^

**Table 2.** Average branch formation on GABAergic dorsal commissures and axons between DD5 and VD5 cell bodies in N2 wild type and *gbb-1* mutant worms (n=50).

**Table:** 

**Day**	**3**	**6**	**9**	**12**	**15**	**18**
***wild type***	0.10 ± 0.58	0.36 ± 0.72	0.2 ± 0.49	0.46 ± 0.76	0.7 ± 1.23	1.02 ± 1.15
***gbb-1* mutant**	0.24 ± 0.66	0.54 ± 1.09	0.96 ± 1.19	1.46 ± 1.86	1.98 ± 2.34	2.3 ± 2.13
**p-value**	0.261	0.333	6.93 x 10^-5*^	6.38 x 10^-4*^	9.19 x 10^-4*^	3.13 x 10^-4*^

## Reagents

The strains used in this study were: CZ10175: *zdIs5[Pmec-4::GFP],* IC2142: *zdIs5[Pmec-4::GFP]; gbb- 1(tm1406),* CZ1197: *juIs73[Punc-25::GFP],* IC2141: *juIs73[Punc-25::GFP]; gbb-1(tm1406)*.
